# The Role of Intestinal Microbiota in Development of Irinotecan Toxicity and in Toxicity Reduction through Dietary Fibres in Rats

**DOI:** 10.1371/journal.pone.0083644

**Published:** 2014-01-14

**Authors:** Xiaoxi B. Lin, Arazm Farhangfar, Rosica Valcheva, Michael B. Sawyer, Levinus Dieleman, Andreas Schieber, Michael G. Gänzle, Vickie Baracos

**Affiliations:** 1 University of Alberta, Department of Agricultural, Food and Nutritional Science, Edmonton, Canada; 2 Department of Oncology, University of Alberta, Edmonton, Alberta, Canada; 3 The Center of Excellence for Gastrointestinal Inflammation and Immunity Research, University of Alberta, Edmonton, Canada; 4 University of Bonn, Department of Nutritional and Food Sciences, Bonn, Germany; Charité-University Medicine Berlin, Germany

## Abstract

CPT-11 is a drug used as chemotherapy for colorectal cancer. CPT-11 causes toxic side-effects in patients. CPT-11 toxicity has been attributed to the activity of intestinal microbiota, however, intestinal microbiota may also have protective effects in CP!-11 chemotherapy. This study aimed to elucidate mechanisms through which microbiota and dietary fibres could modify host health. Rats bearing a Ward colon carcinoma were treated with a two-cycle CPT-11/5-fluorouracil therapy recapitulating clinical therapy of colorectal cancer. Animals were fed with a semi-purified diet or a semi-purified diet was supplemented with non-digestible carbohydrates (isomalto-oligosaccharides, resistant starch, fructo-oligosaccharides, or inulin) in 3 independent experiments. Changes in intestinal microbiota, bacteria translocating to mesenteric lymphnodes, cecal GUD activity, and cecal SCFA production, and the intestinal concentration of CPT-11 and its metabolites were analysed. Non-digestible carbohydrates significantly influenced feed intake, body weight and other indicators of animal health. The identification of translocating bacteria and their quantification in cecal microbiota indicated that overgrowth of the intestine by opportunistic pathogens was not a major contributor to CPT-11 toxicity. Remarkably, fecal GUD activity positively correlated to body weight and feed intake but negatively correlated to cecal SN-38 concentrations and IL1-β. The reduction in CPT-11 toxicity by non-digestible carbohydrates did not correlate to stimulation of specific bacterial taxa. However, cecal butyrate concentrations and feed intake were highly correlated. The protective role of intestinal butyrate production was substantiated by a positive correlation of the host expression of MCT1 (monocarboxylate transporter 1) with body weight as well as a positive correlation of the abundance of bacterial butyryl-CoA gene with cecal butyrate concentrations. These correlations support the interpretation that the influence of dietary fibre on CPT-11 toxicity is partially mediated by an increased cecal production of butyrate.

## Introduction

CPT-11 (irinotecan, 7-ethyl-10-[4-(1-piperidino)-1-piperidino]carbonyloxy-camptothecin) is a drug commonly used as a first-line chemotherapy for colorectal cancer. Therapeutic doses of CPT-11 cause prevalent toxic side-effects in patients. Late onset diarrhea is one of the most common symptoms that limit the application and efficacy of CPT-11, and has been attributed to enzymatic activities of intestinal microbiota. Gastrointestinal symptoms were substantially reduced when antibiotics or inhibitors of bacterial glucuronidase were used in combination with CPT-11 [1], [2]. However, the use of broad spectrum of antibiotics often leads to severe disruption of the microbial homeostasis in the intestine and can result in other negative consequences [3]. A promising alternative of modulating microbiota is administration of dietary fibres, i.e. non-digestible polysaccharides that resist digestion in the small intestine and are fermented by intestinal microbiota in the large intestine. Dietary fibres not only stimulate beneficial bacteria but also to provide short chain fatty acids as an essential substrate for the colonic mucosa, and modulate activities of bacterial enzymes [4]. Therefore, they may ameliorate or mitigate CPT-11 toxicity without causing pronounced side-effects.

**Figure 1:**
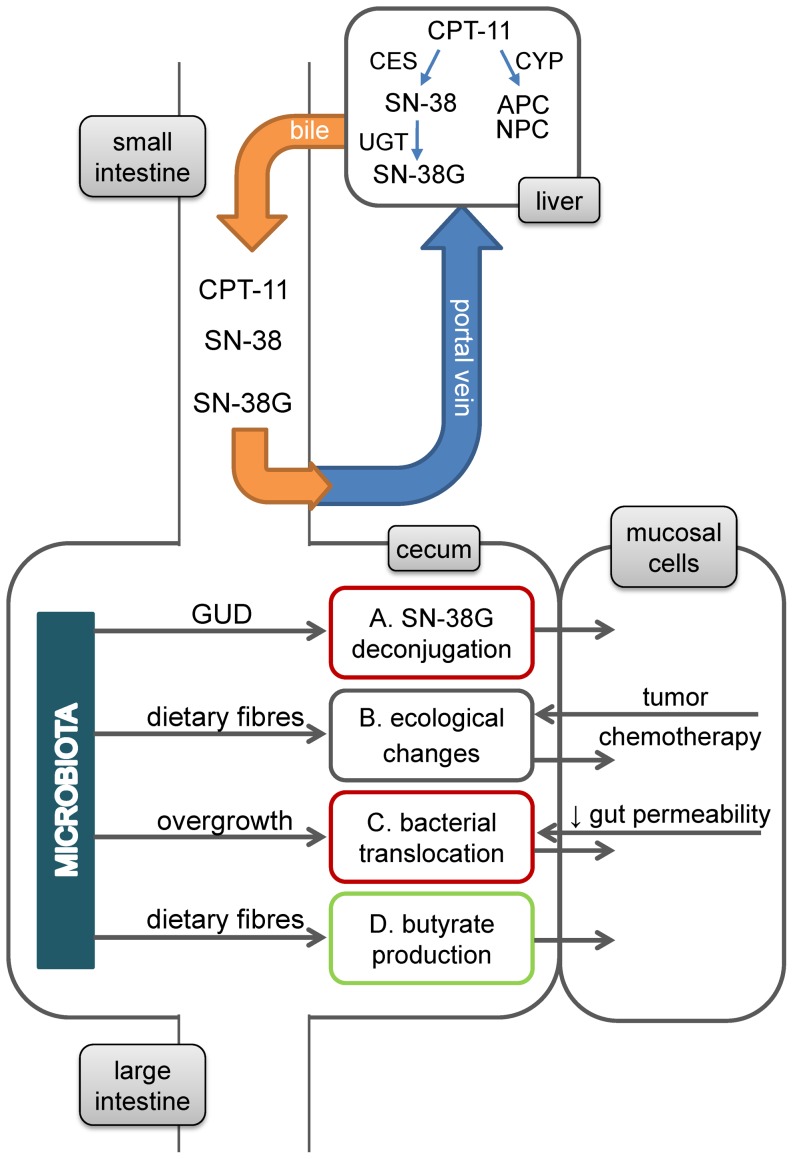
Potential roles of intestinal microbiota in development and mitigation of CPT-11 (irinotecan, 7-ethyl-10-[4-(1-piperidino)-1-piperidino]carbonyloxy-camptothecin) toxicity. *In vivo*, CPT-11 is converted to the pharmacologically active SN-38 (7-ethyl-10-hydroxy-camptothecin), which is responsible for both anti-tumor activity and dose-limiting toxicity. SN-38 undergoes hepatic glucuronidation and is secreted into the bile as inactive the glucuronide SN-38G. Deconjugation of SN-38G in large intestine by bacterial β-glucuronidases intensifies the epithelial exposure to SN-38 and mediates gut toxicity [2], [43], [44]. APC (7-ethyl-10-[4-N-(5-aminopentanoic acid)-1-piperidino]carbonyloxycamptothecin) and NPC (7-ethyl-10-(4-amino-1-piperidino)carbonyloxycamptothecine) are both important CPT-11 metabolites produced by alternative hepatic detoxification mechanism, but they play little role in gastrointestinal toxicity [45]. The composition of intestinal microbiota was reported to be altered in colorectal cancer patients [6], [8] and by various chemotherapeutic agents [7], [9]. On the other hand, dietary fibres are known to promote the growth of beneficial bacteria in animals and human [46]. Bacterial translocation to extraintestinal sites, was reported to be increased by CPT-11 treatment [7], [47] increased the risk of systemic infection. Bacterial translocation from intestine can be promoted by overgrowth of selective species in intestinal microbiota and/or a compromised host defense system, including immunodeficiency and increased intestinal permeability [11], both of which have been reported in CPT-11-treated animals [7], [48]. Dietary fibres have been shown to promote intestinal health through bacterial fermentation products short-chain fatty acids (SCFA), especially butyrate. The majority of butyrate is consumed by mucosal cells. Its main functions in intestine include (1) promoting proliferation and growth of normal colonocytes, (2) enhancing epithelial barrier function, and (3) suppressing inflammation and oxidative stress [36], [42] Therefore, the protective effect of butyrate may counteract injuries caused by SN-38.

Figure 1 illustrates the potential roles of intestinal microbiota in CPT-11 toxicity. A major player in the pharmacokinetics of CPT-11 is microbial β-glucuronidase (GUD), which deconjugates the CPT-11 metabolite SN-38G to regenerate the toxic metabolite SN-38 in the large intestine. Microbial β-glucuronidase therefore is considered to be responsible for CPT-11-associated gut damage (Figure 1); intestinal injury and shifts in intestinal microbiota further facilitate bacterial translocation (Figure 1). However, microbiota can also positively affect host health through SCFA (especially butyrate) production. Bacterial groups differ in their contribution to these potential mechanisms. *Bifidobacterium* spp., *Enterobactericeae, Bacteroides* spp., *Lactobacillus* spp., *Staphylococcus* spp., and species from *Clostridium* Cluster XIVa and IV exhibit GUD activity [5]. Intestinal dysbiosis can be induced by both tumor and chemotherapy. Dysbiosis associated with cancer was characterized by an increase in *Enterobacteriaceae* and decrease in butyrl-CoA producing bacteria [6]. In dysbiosis caused by various chemotherapies, increase in *Escherichia coli*, *Clostridium* spp., and *Staphylococcus* spp. and decrease in *Bifidobacterium* spp. and *Bacteroides* spp. were reported [7], [8], [9]. Bacterial species implicated in bacterial translocation were mostly facultative anaerobes and opportunistic pathogens, including *Enterococcus* spp., *Streptococcus* spp., *Staphylococcus* spp., and *Enterobacteriaceae* [10], [11]. Butyrate, the primary energy source for colonocytes, is mainly produced by *Clostridium* cluster IV and XIVa [12].

The multitude of potential involvements of microbiota in CPT-11 toxicity make CPT-11 treated animals a unique model for investigating the interaction between microbiota and host. This study aimed to explore the potential mechanisms through which microbiota and dietary fibres could modify host health: changes in intestinal microbial ecology, translocation, GUD activity, and SCFA production.

## Methods

### Animals and treatments

Animal use was approved by the Animal Care and Use Committee of the University of Alberta and conducted in accordance with Guidelines of the Canadian Council on Animal Care (AC08153). Female Fisher 344 rats (150–180 g of body weight and 11–12 weeks of age) were obtained from Charles River (QC, Canada). The use of female animals avoided potential confounding effects of sex, and allowed direct comparison with prior work in the same tumor model [13], [14]. Rats were housed 2 per cage in a temperature (22°C) and light controlled (12 h light) room; water and food were available *ad libitum*. One week before chemotherapy, rats were separated into individual cages. The Ward colon carcinoma was provided by Dr. Y Rustum, Roswell Park Institute. Tumor pieces (0.05 g) were transplanted subcutaneously on the flank of the rats via trocar under slight isoflurane anesthesia. CPT-11 was provided by Pfizer as a ready-to-use clinical formulation. Atropine (0.6 mg/ml) was a clinical injectable formulation. Rats were killed by CO_2_ asphyxiation.

### Diet

Diets used in this study are described elsewhere [13]. Briefly, semi-purified diet was based on AIN-76 basal diet, with a modified fat component similar to a North American dietary pattern with respect to energy % as fat and levels of n-3, n-6, saturated and polyunsaturated fatty acids. Rats were initially fed Rodent Laboratory Chow (Harlan Teklad, Madison, WI). During the adaptation period, this non-purified diet was mixed with study diet (50/50, w/w) for one week, followed by transition to 100% semi-purified diet starting 2 weeks prior to tumor implantation.

### Experimental design

**Figure 2:**
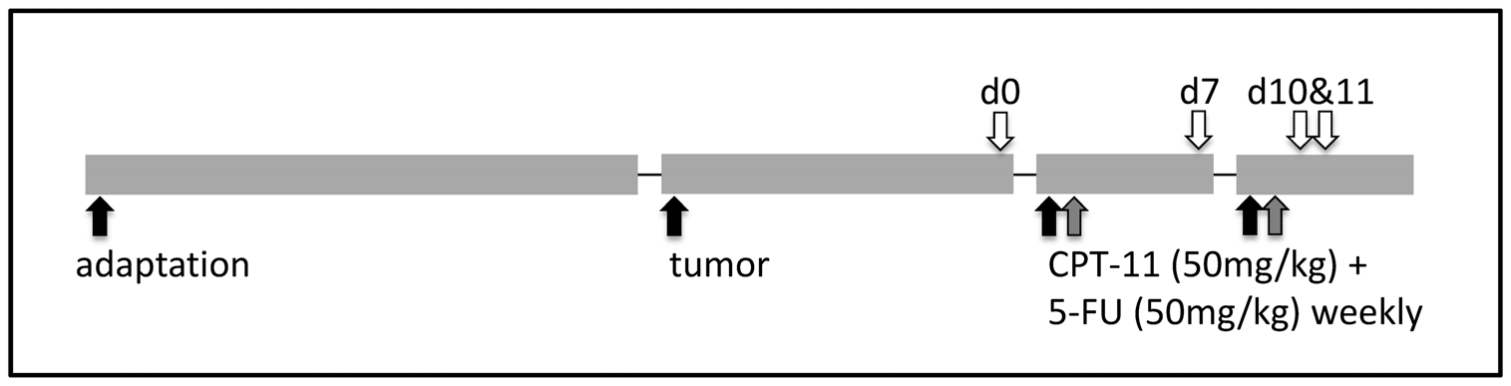
Experimental design. Black arrows represent diet, tumor, and CPT-11 treatments; grey arrows represent treatment with 5FU. Sampling at day 10 and 11 after the first CPT-11 treatment is indicated by white arrows.

All experiments used a two-cycle CPT-11/5-fluorouracil (5-FU) therapy which recapitulates clinical therapy of colorectal cancer (Figure 2). Rats received two cycles of CPT-11/5-FU treatment. The day before first CPT-11 injection was designated day 0. Animals received CPT-11 (50 mg/kg) and 5-FU (50 mg/kg) injections on days 1 and 8 and on days 2 and 9, respectively. **Exp 1** was designed to investigate the influence of chemotherapy on intestinal microbiota and bacterial translocation [9]. Animals (n = 6/group) were killed on day 0, day 7 (prior to the second treatment cycle), and days 10 and 11 (one and two days after the 2nd treatment cycle). **Exp 2** was designed to evaluate and compare the effect of dietary fibres on CPT-11 toxicity. Animals (n = 6/group) received diets containing the following dietary fibres: commercial isomalto-oligosaccharides (IMO); fructooligosaccharides (FOS); inulin; a 1∶1 mixture of FOS and inulin (synergy); and Type IV resistant starch (RS). These fibre sources differing in their structure and/or degree of polymerization were incorporated into the modified AIN-76 basal diet at 8% w/w. The dietary composition is shown in the online supplemental Table S1. A seventh diet where the fibres were substituted with equal amount of starch was used as control. All diets contained 2% w/w cellulose. **Exp 3** was designed to compare two different dietary fibres with larger sample size. IMO and synergy were selected as the treatment diet. Cellulose, which has the lowest fermentability in rat intestine, was used as control. Healthy rats without tumor or chemotherapy were used as reference. Sample size was 14 animals per treatment for synergy and IMO diets and 6 per treatment for healthy control and cellulose diet. In experiments 2 and 3, animals were killed at day 9 as chemotherapy-induced morbidity and mortality become apparent at this time [14].

### Cecal gene expression of monocaboxylate transporter 1 (MCT1), glutathione S-transferase (GSTP1), and mucin genes

Gene expression was quantified using cecal tissue collected in Exp 3. Tissues were stored in Trizol® (Invitrogen) at −80°C. For RNA extraction, tissues were thawed in Trizol and homogenized using Kontes Pellet Pestle (Fisher Scientific). The homogenized solution was mixed with 20% v/v of chloroform and centrifuged for 15 min at 4°C. The supernatant mixed with an equal volume of 70% ethanol and applied to the column from Qiagen Rneasy Mini Kit (Qiagen, Mississauga, Canada). RNA was purified following the manufacturer's instructions and then reverse-transcribed to cDNA using QuantiTect Reverse Transcription Kit (Qiagen). Relative gene expression was calculated as (E*_target_*)^ΔCP(control-sample)^/(E*_reference_*)^ΔCP(control-sample)^ [15]. Expression of MCT1, GSTP1, and the mucin genes MUC1 and MUC2 were quantified using the ubiquitin c gene *ubc* as reference gene due to its stable expression in normal and CPT-11-treated rats [16], [17]. Healthy rats fed a 10% cellulose diet were used as control. Primers used were: *gstp1*, forward: 5′-GAT GGG GTG GAG GAC CTT CGA TGC-3′, reverse: 5′-CTG AGG CGA GCC ACA TAG GCA GAG-3′ [18]; *mct1*, forward: 5′-CAG TGC AAC GAC CAG TGA AGT G-3′, reverse: 5′-ATC AAG CCA CAG CCA GAC AGG-3′ [19]; *ubc*, forward: 5′-ATC TAG AAA GAG CCC TTC TTG TGC-3′, reverse: 5′-ACA CCT CCC CAT CAA ACC C-3′ [17]; *muc1*, forward: 5′-CGC CGA AAG AGC TAT G-3′, reverse: 5′-TAA GAG AGA CCG CTA CTG CC-3′ [2]; *muc2*, forward: 5′-GCC AGA TCC CGA AAC CA-3′ and reverse: 5′- TAT AGG AGT CTC GGC AGT CA-3′ [2]; IL-1β, forward: 5′-GCA CCT TCT TTT CCT TCA TC-3′ and reverse: 5′-CTG ATG TAC CAG TTG GGG AA-3′; TNF-α, forward: 5′-GGC AGG TCT ACT TTG GAG TCA TTG C-3′, reverse: 5′-ACA TTC GGG GAT CCA GTG AGT TCC G-3′ [3]. Quantitative PCR was performed on 7500 Fast Real-Time PCR (Applied Biosystem) using SYBR Green reagents (Qiagen). DNase-treated RNA samples were also amplified as negative controls to ensure the quality of reverse transcription.

### DNA extraction and quantification of major bacterial groups by quantitative PCR (qPCR)

DNA was extracted from digesta samples using the QIAamp DNA Stool Mini Kit (Qiagen). Quantitative PCR was performed. Major bacterial groups in cecal and fecal microbiota were quantified using group-specific primers targeting total bacteria, *Bacteroides-Prevotella-Porphyromonas* (*Bacteroides* group), *Lactobacillus-Pediococcus-Leuconostoc-Weissella (Lactobacillus* group), *Bifidobacterium* spp., *Clostridium* clusters I, IV, XI, and XIV, *Enterobacteriacaea* [5], *Ruminococcus gnavus* [6], and *Akkermansia muciniphila* [7]. Diarrhea- and enteric infection-associated virulence factors in cecal microbiota were quantified using primers described previously targeting virulence factor/toxin genes of enteropathogenic *Clostridium difficile* (tcdB) and *E. coli* (STa, STb, LT, EAST1) [5].

### Identification of bacterial species carrying GUD genes in cecal microbiota

Cecal DNA in Exp 2 were amplified with primers targeting the bacterial GUD *gus* gene (forward: 5′-TAT TTA AAA GGI TTY GGI MRI CAY GAR-3′, reverse: 5′-CCT TCT GTT GTI KBR AAR TCI GCR AAR-3′) [20]. PCR amplification was performed using the following conditions: initial denaturation (3 min at 94°C), then 35 cycles of denaturation (30 s at 94°C), ramped annealing (20 s at 55°C, 5 s at 50°C, and 5 s at 45°C), and elongation (1 min at 72°C) and a final extension (7 min at 72°C). PCR products were cloned with TOPO TA Cloning kit (Invitrogen) according to the manufacturer's instructions. Sequencing was done at Macrogen Inc. with vector primers M13F (5′-GTA AAA CGA CGG CCA G-3′) and M13R (5′-GGAAACAGCTATGAC-3′) and sequences were identified by comparing to the known *gus* genes in the GenBank database using BLAST.

### Quantification of cecal lactate and SCFA concentrations

Cecal samples from Exp 2 and 3 were incubated with 7.5% perchloric acid at 4°C overnight to remove proteins. Lactate and SCFA were separated using an Aminex 87H column (Bio-Rad, Mississauga, Ontario) at a temperature of 70°C, and the solvent was 5 mM H_2_SO_4_, at a flow rate of 0.4 mL/min. Metabolites were visualized using a UV detector at 210 nm, and quantified using external standards.

### Quantification of haptoglobin and acute phase proteins

For quantification of haptoglobin and acute phase proteins in blood, commercial ELISA kits for rat α-1-acid glycoprotein (AGP) and rat haptoglobin were purchased from Life Diagnostics (West Chester, Pennsylvania, USA) and used according to the manufacturer's instructions [4].

### Quantification of CPT-11 metabolites in cecum and jejunum digesta

Cecal digesta from Exp 2 and cecal and jejunum digesta from Exp 3 were analyzed for CPT-11 and its metabolites. The digesta were extracted 3 times with 67% methanol overnight and solids were removed by centrifugation. The supernatant was then diluted 6 times with water. Metabolite concentration was quantified using a 4000 QTRAP® LC/MS/MS System (AB Sciex, Canada) following a protocol by Corona et al. [21] with slight modifications. A Kinetex™ 2.6 µm C18 100×3 mm column (Phenomenex, Canada) was used. Assays were performed using LC-MS/MS under ESI-MRM mode. The flow rate was 0.4 mL/min. The acetonitrile gradient increased from 10% to 80 % from 0 to 18 min, maintained at 100% from 18.1 to 19.0 min, and returned to 10% from 19.4 to 25 min. MRM transitions used were: *m/z* 587→167 for CPT-11, *m/z* 393→349 for SN-38 (7-ethyl-10-hydroxy-camptothecin), *m/z* 619→393 for APC (7-ethyl-10-[4-N-(5-aminopentanoic acid)-1-piperidino]carbonyloxycamptothecin), *m/z* 519→393 for NPC (7-ethyl-10-(4-amino-1-piperidino)carbonyloxycamptothecine), and *m/z* 569→393 for SN-38-glucuronide (SN-38G). Quantification was done with standards prepared from pure CPT-11, APC, NPC, SN-38, SN-38G purchased from Tocris Bioscience, UK.

### Isolation and identification of bacteria from mesenteric lymph nodes

Bacterial translocation to mesenteric lymph nodes was determined with samples obtained in Exp 1. Isolates from mesenteric lymph nodes were obtained by serial dilution streaking on sheep blood agar plates and incubation at 37°C for 48 h under aerobic conditions. Isolates were subsequently cultured in Brain Heart Infusion medium at 37°C. DNA was extracted from overnight cultures using DNeasy Blood, Tissue Kit (Qiagen, USA). Bacterial 16S rRNA genes were amplified using 616V (5′ –AGA GTT TGA TYM TGG CTC-3′) and 630R (5′-CAK AAA GGA GGT GAT CC-3′) universal primers, and sequenced at Macrogen Corp. (MD, USA). For identification of isolates on genus or species level, sequences were matched to sequences of type strains deposited in the Ribosomal Database Project (rdp.cme.msu.edu).

### Statistical analysis

Statistical analysis was performed with PROC MIXED procedure (SAS v.9.2; SAS Institute, 2010) using one-way analysis of variance (ANOVA). Associations between variables were analyzed using Spearman correlation. Variables with non-parametric distribution were log-transformed prior to analysis. A p-value of ≤0.05 was considered statistically significant.

## Results

### The diet effect on animal health was not dependent on the type of fibre

In all three experiments, animals experienced weight loss and a significant reduction in feed intake following each chemotherapy cycle, with the effect of the second cycle being more pronounced [14], [22]. Addition of non-digestible carbohydrates significantly influenced feed intake, body weight and other indicators of animal health [22]. However, all treatment groups displayed large intra-group variation indicating that factors other than the diet also had a major influence on animal health. The six different fibres used in Exp 2 resulted in a range of outcomes in rats: animals fed on RS and Synergy had a significantly lower weight loss and reduction in feed intake compared to those fed on starch and IMO [22]. In Exp 3, healthy rats without tumor and not receiving chemotherapy had much higher body weight and food intake than chemotherapy-treated rats.

### Overgrowth of the intestine by opportunistic pathogens is not a major contributor to CPT-11 toxicity

**Figure 3:**
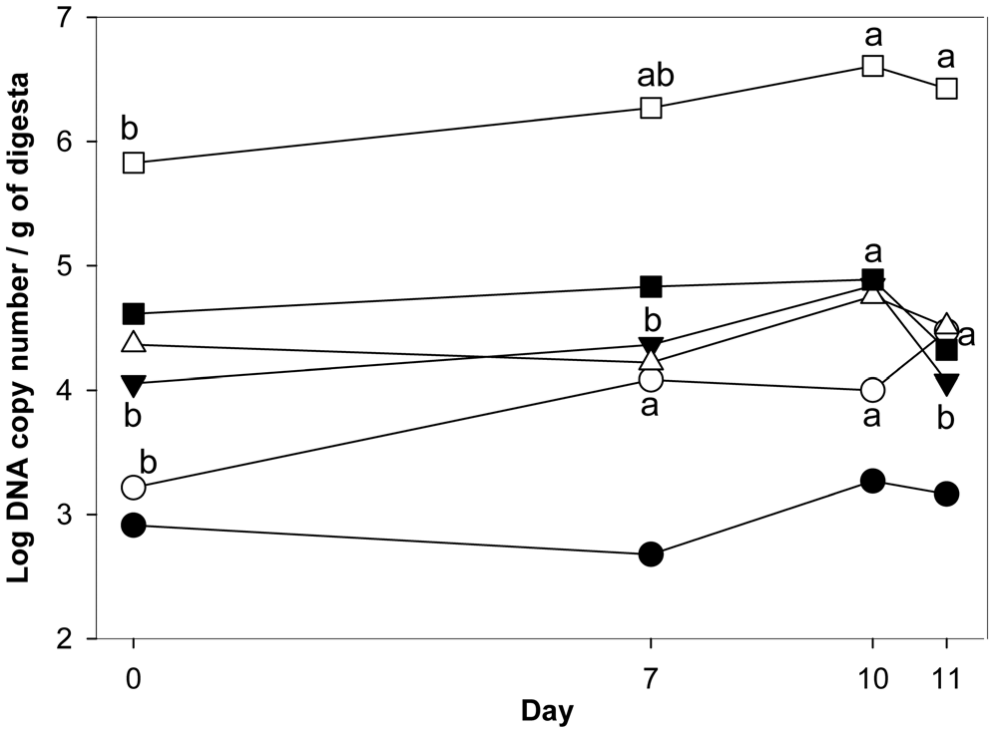
Gene copy numbers for major bacterial groups per gram of cecal digesta at day 0, 7, 10, and 11 in Exp 1. Symbols indicate *Proteus mirabilis* (•), *Citrobacter freundii* (**○**), *Klebsiella oxytoca* (▾), *Escherichia coli* (Δ), *Staphylococcus* spp. (▪), and *Enterococcus* spp. (□). Data are shown as mean±SEM. Values obtained with the same primers that do not share a common letter differ significantly; i.e. vary over time.

Mesenteric lymph nodes from healthy animals were sterile. Bacteria in mesenteric lymph nodes from chemotherapy treated animals (Exp 1 and 2) were isolated and identified by partial sequencing of 16S rRNA genes. Isolates from infected mesenteric lymph nodes belonged to the family *Enterobacteriaceae* and the genera *Enterococcus* and *Staphylococcus* (Table S2 of the online supplementary material). To determine whether translocation was favoured by a higher abundance of these opportunistic pathogens in the gut lumen, bacterial taxa representing translocated bacteria were quantified in cecal digesta by qPCR (Figure 3). *Morganella morganii* was below the detection limit in all samples. An increased abundance of *Citrobacter freundii, Klebsiella oxytoca*, and *Enterococcus* spp. following chemotherapy was observed, but the abundance of other translocated organisms remained unchanged (Figure 3). Despite unfavourable changes in intestinal microbiota during chemotherapy, compromised barrier properties of the gut mucosa as a result of CTP-11 toxicity rather than overgrowth of the intestine by opportunistic pathogens appears to be the primary cause of bacterial translocation.

### Effect of fibres on intestinal microbiota and host health: Experimental design and data analysis

**Figure 4:**
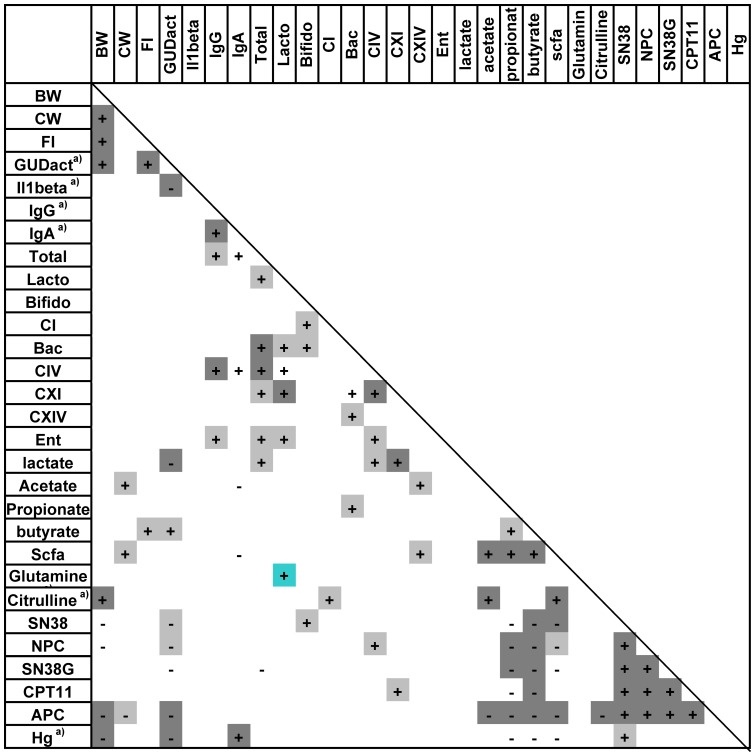
Pixel mapping highlights of major microbiota-host correlations of Exp 2. + indicates a positive correlation; - indicates negative correlation. Spearman correlation p-values are displayed as a greyscale, dark grey, p<0.005; light grey, p<0.05; no shading, trend (p<0.1). Key: BW, relative body weight; CW, colon weight; FI, cumulative food intake; Total, total bacteria; Lacto, *Lactobacillus* group; Bifido, *Bifidobacterium* spp.; CI, *Clostridium* cluster I, Bac, *Bacteroides* group; CIV, CXI, CXIV, *Clostridium* clusters IV, XI, and XIV; Ent, *Enterobacteriacaea*; scfa, short chain fatty acids; hg, haptoglobin.

**Figure 5:**
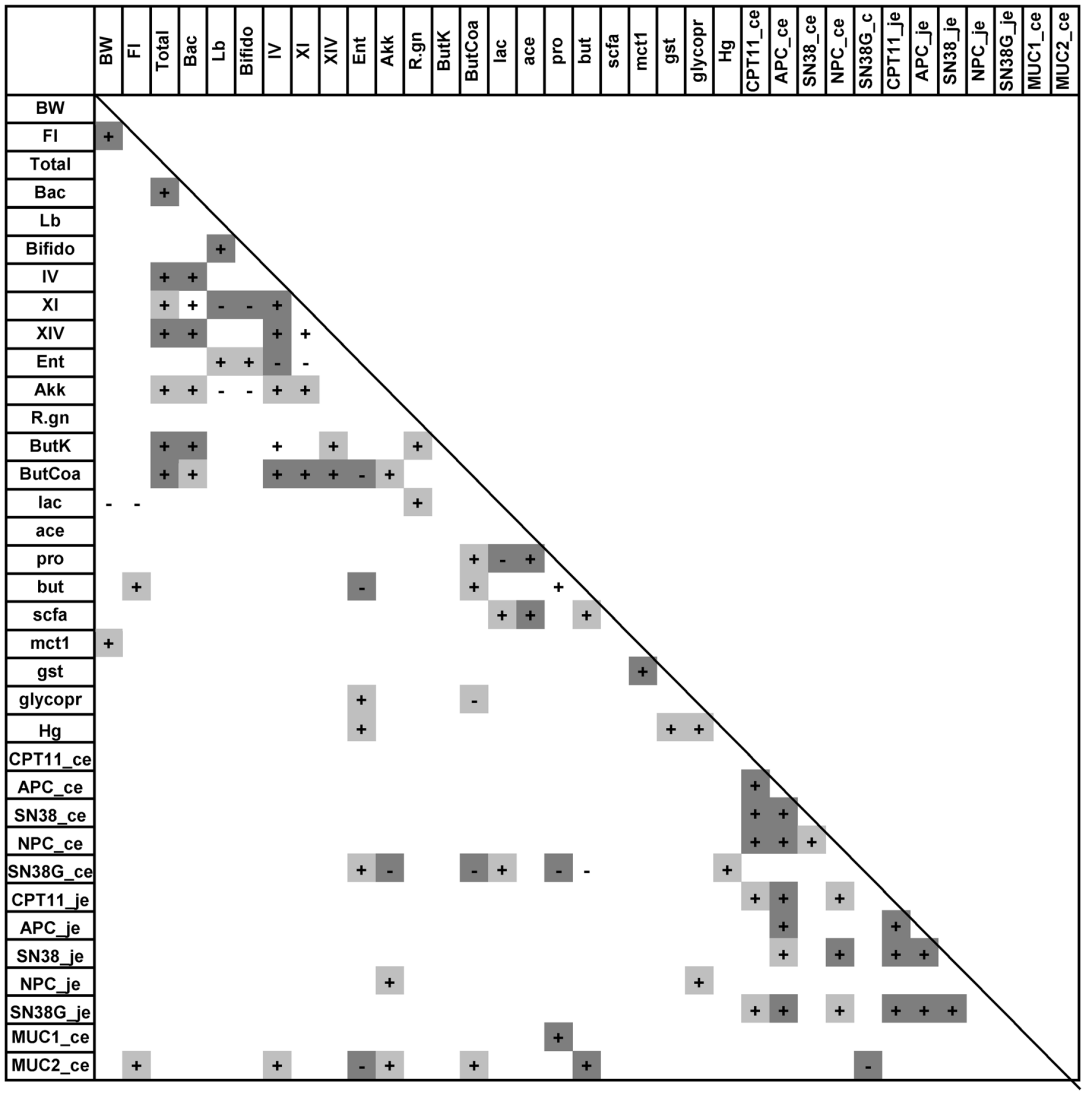
Pixel mapping highlights of major microbiota-host correlations of Exp 3. Spearman correlation p-values are displayed as a greyscale, dark grey, p<0.005; light grey, p<0.05; no shading, trend (p<0.1). Key: BW, relative body weight; FI, cumulative food intake; Total, total bacteria; Bac, *Bacteroides* group; Lb, *Lactobacillus* group; Bifido, *Bifidobacterium* spp.; CIV, CXI, CXIV, *Clostridium* clusters IV, XI, and XIV; Ent, *Enterobacteriacaea*; Akk, *Akkermansia*; R. gn. *Ruminococcus gnavus*; lac, lactate; ace, acetate; pro, propionate; but, butyrate; scfa, short chain fatty acids; mct1, relative expression of monocarboxylate transporter 1; gst, relative expression of glutathione-S-transferase; glycopr, blood glycoprotein; hg, blood haptoglobin; CPT11_ce and CPT11_je, cecal and jejunal concentration of CPT-11, respectively; APC_ce and APC_je, cecal and jejunal concentration of APC, respectively; SN38_ce and SN38_je, cecal and jejunal concentration of SN38, respectively; NPC_ce and NPC_je, cecal and jejunal concentration of NPC, respectively; SN38G_ce and SN38G_je, cecal and jejunal concentration of SN38-G, respectively; MUC1_ce and MUC2_ce, relative expression of *muc1* and *muc2*, respectively, in cecal tissue.

The effect of dietary fibre on intestinal microbiota and host health was assessed in two experiments. An initial, explorative experiment compared six different purified fibre types with minimal statistical power (N = 6). A second experiment compared to purified fibre types, IMO and synergy, with a higher number of animals per group (n = 14). Intestinal and host parameters for experiments 2 and 3 are reported by diet in tables 1 and 2, respectively. Because differences between diets were not greater than the differences between animals in the same group, results relating to host health and intestinal microbiota were also correlated to each other after pooling all animals used in experiment 2 and experiment 3. The results are depicted in heat maps (Figures 4 and 5, respectively).

**Table 1:** Abundance of major bacterial groups and SCFA concentration per gram of cecal digesta in Exp 2.

		Starch	IMO	Cellulose	FOS	Inulin	Synergy	RS
**Total bacteria**	copy number/g of cecal digesta	9.0 (0.4)	8.8 (0.3)	8.7 (0.3)	9.1 (0.3)	8.8 (0.6)	9.2 (0.3)	9.3 (0.3)
***Bacteroides*** ** group**		9.6^ab^ (0.4)	9.6^ab^ (0.4)	9.5^b^ (0.1)	10.0^a^ (0.3)	9.7^ab^ (0.4)	9.8^ab^ (0.2)	10.0^ab^ (0.4)
***Lactobacillus*** ** group**		8.1^a^ (0.4)	7.9^ab^ (0.5)	7.4^b^ (0.1)	7.8^ab^ (0.7)	7.6^ab^ (0.5)	7.8^ab^ (0.1)	8.3^a^ (0.4)
***Bifidobacterium*** ** spp.**		6.0 (5.7–6.1)	5.7 (4.7–6.0)	5.7 (5.6–6.0)	5.8 (5.4–7.8)	5.9 (4.5–6.4)	5.8 (5.6–6.0)	5.8 (4.9–6.3)
**Cl. cluster I**		6.3 (0.5)	6.0 (0.5)	5.8 (0.3)	6.6 (0.6)	6.7 (1.0)	6.2 (0.8)	6.1 (0.4)
**Cl. cluster IV**		8.6 (0.6)	8.2 (0.5)	8.1 (0.4)	8.2 (0.6)	8.0 (0.6)	8.5 (0.5)	8.7 (0.4)
**Cl. cluster XI**		4.9 (4.6–5.8)	4.8 (4.0–6.1)	4.4 (4.2–5.0)	4.7 (3.6–5.4)	3.8 (3.5–5.0)	4.7 (3.7–5.3)	4.8 (4.3–5.3)
**Cl. cluster XIVa**		8.1 (7.8–8.9)	8.7 (7.5–8.9)	8.1 (7.8–9.8)	8.7 (8.0–9.3)	8.6 (7.5–9.0)	9.0 (7.8–9.7)	8.8 (8.4–8.9)
***Enterobacteriaceae***		5.4 (0.8)	4.1 (0.2)	3.9 (0.2)	4.5 (0.8)	4.6 (1.0)	4.3 (0.5)	4.2 (0.3)
**Lactate**	µmol/g of cecal digesta	11.5 (6.16–23.1)	10.9 (7.54–14.9)	8.47 (6.34–10.7)	8.28 (5.22–11.1)	6.32 (4.37–8.03)	11.3 (7.27–19.9)	7.46 (4.05–19.0)
**Acetate**		32.7 (7.35)	24.1 (5.81)	28.4 (4.03)	37.0 (9.73)	30.6 (3.40)	30.5 (7.70)	28.7 (8.77)
**Propionate**		6.42 (1.30)	5.77 (0.86)	5.52 (1.17)	7.70 (2.44)	6.54 (0.49)	6.70 (0.66)	7.34 (1.65)
**Butyrate**		4.02 (1.64–6.87)	3.35 (1.04–6.93)	3.21 (2.21–4.00)	8.51 (2.02–18.0)	10.7 (2.92–24.7)	4.19 (2.58–5.82)	7.33 (3.01–10.6)
**CPT-11**	µg/g cecal digesta	86.3 (35.2–123.0)	55.0 (15.1–93.2)	43.6 (27.1–68.1)	25.5 (10.4–8.5)	32.9 (9.55–84.2)	25.5 (12.6–62.3)	29.7 (21.9–88.1)
**SN-38**		13.9 (5.2–23.0)	7.83 (3.09–13.8)	9.29 (2.35–11.8)	3.70 (0.93–5.55)	7.10 (3.65–18.5)	4.70 (1.68–8.48)	6.01 (2.60–20.0)
**APC**		9.02 (2.64–21.9)	18.8 (2.40–113.4)	8.47 (6.41–14.3)	3.68 (1.94–6.25)	5.55 (2.52–17.9)	4.02 (2.14–7.79)	3.74 (2.29–113.4)
**NPC**		0.23 (0.063–0.25)	0.062 (0.023–0.141)	0.093 (0.048–0.164)	0.038 (0.015–0.079)	0.046 (0.013–0.119)	0.039 (0.021–0.081)	0.049 (0.026–0.386)
**SN-38G**		0.041(0.007–0.138)	0	0.047 (0.009–0.101)	0	0	0	0

Values within brackets are 95% confidence intervals for variables with parametric distribution and 5% and 95% percentiles for variables with non-parametric distribution. Values within a row without superscripts are not significantly different. Values without superscripts are not significantly different from any other value in the same row. Values within a row that do now share a common superscript are significantly different (P<0.05).

**Table 2:** Abundance of major bacterial groups, stress response, MCT1 expression, SCFA concentration, and CPT-11 metabolite concentrations per gram of cecal digesta in Exp 3.

		IMO	SYN	CEL	Ref
Relative body weight		0.91 (0.01)^b^	0.92 (0.01)^b^	0.93 (0.02)^ab^	1.00 (0.02)^a^
Relative food intake		0.73 (0.07)^b^	0.76 (0.07)^b^	0.78 (0.014)^b^	0.99 (0.07)^a^
Total bacteria	Gene copy number/g of cecal digesta	10.7 (0.1)	10.7 (0.1)	10.7 (0.2)	10.6 (0.3)
*Bacteroides* group		9.9 (0.1)	9.8 (0.2)	9.8 (0.3)	9.4 (0.2)
*Lactobacillus* group		8.5^b^ (0.2)	9.1^a^ (0.3)	8.9^ab^ (0.5)	9.0^ab^ (0.3)
*Bifidobacterium* spp.		5.7^c^ (0.3)	7.2^a^ (0.3)	6.4^b^ (0.5)	6.9^ab^ (0.4)
*Clostridium* cluster IV		8.4 (7.9–8.7)	8.0 (6.3–8.8)	8.5 (7.3–8.8)	8.8 (8.2–8.8)
*Clostridium* cluster XI		5.2^b^ (0.0–6.0)	0.0^c^ (0.0–1.9)	4.8^b^ (1.1–5.5)	5.6^a^ (5.4–5.8)
*Clostridium* cluster XIVa		10.0 (0.1)	10.1 (0.2)	10.0 (0.2)	10.2 (0.4)
*Enterobacteriacea*		8.2 (0.3)	8.8 (0.6)	7.8 (0.7)	7.4 (1.0)
*Akkermansia muciniphila*		8.5 (0.3)	8.0 (0.2)	7.7 (0.6)	8.6 (0.5)
*Ruminococcus gnavus*		6.8 (0.4)	7.0 (0.3)	6.4 (0.8)	7.1 (0.3)
Butyrate kinase		6.3 (0.4)	6.3 (0.3)	6.1 (0.6)	5.4 (0.1)
Butyryl-CoA transferase		5.8 (0.2)	5.3^b^ (0.5)	5.6 (0.8)	6.4^a^ (0.3)
Lactate	µmol/g of cecal digesta	5.6 (0.3–13.6)	6.1 (0.6–33.2)	4.6 (3.8–6.3)	5.2 (0.9–6.4)
Acetate		19.4 (1.4)	21.7 (4.1)	19.6 (2.0)	22.1 (1.2)
Propionate		3.6 (0.6)	3.5 (1.8)	3.6 (0.3)	3.4 (0.8)
Butyrate		5.5^b^ (2.9–10.1)	5.3^b^ (2.1–22.4)	7.4^ab^ (4.6–11.0)	16.0^a^ (10.2–19.9)
MCT1		0.33^b^ (0.10)	0.43^ab^ (0.15)	0.72^ab^ (0.15)	1.16^a^ (0.49)
Haptoglobin	g/L	1.611^a^ (0.417)	1.847^a^ (0.366)	1.428^ab^ (0.471)	0.477^b^(0.113)
GSTP1	Fold change	0.70 (0.28)	0.63 (0.24)	0.77 (0.25)	1.09 (0.37)
TNF-α		0.82 (0.09–1.94)	1.12 (0.24–9.83)	0.58 (0.14–1.07)	1.15 (0.59–1.71)
IL1-β		1.38 (0.42–2.67)	1.98 (0.59–7.07)	3.14 (1.05–9.97)	1.16 (0.76–1.22)
MUC1		2.33 (1.16–13.63)	5.77 (2.6–18.26)	2.08 (1.05–3.56)	
MUC2		2.20 (1.59)	1.85 (0.94)	2.01 (0.58)	
CPT-11	µg/g of cecal digesta	22.0 (8.77–80.4)	24.7(4.89–75.9)	22.7 (9.99–56.9)	
SN-38		3.52^a^ (0.04–9.50)	0.80^b^ (0.12–11.2)	2.44^ab^ (0.56–6.54)	
SN-38G		0.02^ab^ (0.02)	0.07^a^ (0.06)	0.0^b^ (0.0)	
APC		1.90 (1.06–5.93)	1.76 (0.89–9.63)	2.35 (0.75–7.64)	
NPC		0.07 (0.02–0.24)	0.04 (0.02–0.13)	0.04 (0.03–0.12)	
CPT-11	µg/g of jejunum digesta	4.88 (1.43–10.5)	3.34 (0.25–9.25)	5.14 (3.79–9.31)	
SN-38		0.19 (0.09–1.27)	0.15 (0.02–0.55)	0.43 (0.31–0.61)	
SN-38G		17.4 (3.23–39.6)	10.8 (0.77–34.6)	16.77 (11.6–34.8)	
APC		0.48 (1.44–10.49)	0.37 (0.04–2.84)	1.24 (0.64–2.38)	
NPC		0.03 (0–0.05)	0.00 (0.00–0.04)	0.03 (0–0.09)	

Values within brackets are 95% confidence intervals for variables with parametric distribution and 5% and 95% percentiles for variables with non-parametric distribution. Values without superscripts are not significantly different from any other value in the same row. Values within a row that do now share a common superscript are significantly different (P<0.05).

### Cecal GUD activity

Chemotherapy treated animals with a high body weight and feed intake surprisingly also had high intestinal GUD activity [22]. Correlation analysis confirmed that fecal GUD activity was positively correlated to body weight and feed intake but negatively correlated to the inflammation marker IL1-β (Figure 4). Protective effects of dietary fibre on CPT-11 toxicity were thus not mediated by reduced activity of intestinal GUD. To further elucidate roles of bacterial GUD, concentrations of CPT-11 and its metabolites were quantified in the cecum and the jejunum (Tables 1 and 2). Concentrations of CPT-11 and its metabolites varied over a wide range. Of the CPT-11 metabolites, SN-38G was present in the highest concentrations in the jejunum. Conversely, SN-38 was present in the highest concentrations in the cecum, where SN-38G was essentially absent (Tables 1 and 2). These data conform to the established role of bacterial GUD in CPT-11 toxicity, and additionally demonstrate that modulation of intestinal GUD activity by dietary intervention does not significantly reduce conversion of SN-38G to the toxic SN-38 in the cecum. Intestinal concentrations of CPT-11 metabolites were positively correlated to each other and to CPT-11 (Figures 4 and 5) but were not significantly related to body weight and feed intake (Figure 4 and 5). Interestingly, GUD activity was negatively correlated to cecal SN-38 concentrations (Figure 4), suggesting that deconjugation of SN-38G by GUD was not the main factor determining levels of SN-38 exposure.

To identify bacterial species responsible for GUD activity, cecal DNA in Exp 2 was amplified with degenerate primers specific for bacterial GUD and amplicons were sequenced (Table 3). In total, 23 out of 98 sequences were positively identified as GUD. two groups that were identified most frequently were *Ruminococcus gnavus* (8 out of 23) from *Clostridium* cluster XIVa, and *Enterobacteriaceae* (6 out of 23) which included the species *Edwardsiella*, *Escherichia*, and *Edwardsiella*.

**Table 3:** Genera of bacterial GUD producers.

Bacterial genus	Family, group or phylum	E-value	Max identa)
Several unrelated genera	*Firmicutes*	9.1	89%
*Frankia*	*Actinobacteria*	1.1	92%
*Sulfolobus*	Archae	4.21	100%
*Bacteroides*	*Bacteroides*	0.009	51%
*Bifidobacterium*	*Bifidobacterium*	8.0E-13	76%
*Clostridium*	*Clostridum* cluster I	0.003	77%
*Faecalibacterium*	*Clostridum* cluster IV	4.0E-125	74%
*Ruminococcus* b)	*Clostridum* cluster XIV	0.12	84%
*Ruminococcus*	*Clostridum* cluster XIV	2.6	87%
*Ruminococcus*	*Clostridum* cluster XIV	0.16	88%
*Ruminococcus*	*Clostridum* cluster XIV	2.1	87%
*Ruminococcus*	*Clostridum* cluster XIV	0.029	91%
*Ruminococcus*	*Clostridum* cluster XIV	2.6	87%
*Ruminococcus*	*Clostridum* cluster XIV	0.35	88%
*Ruminococcus*	*Clostridum* cluster XIV	0.16	88
*Edwardsiella*	*Enterobacteriaceae*	1.3	100%
*Edwardsiella*	*Enterobacteriaceae*	0.16	100%
*Edwardsiella*	*Enterobacteriaceae*	0.082	100%
*Edwardsiella*	*Enterobacteriaceae*	0.62	90%
*Escherichia* ^b^	*Enterobacteriaceae*	1.1	87%
*Escherichia* b)	*Enterobacteriaceae*	3.3	87%
*Lactobacillus*	*Lactobacillaceae*	7.5	85%
*Staphylococcus*	*Staphylococcaceae*	1.3	90%

^a)^ the minimum length of sequence data considered in the analysis was 200 bp.

^b)^ Sequences attributed to *Ruminococcus* spp. all matched to *R. gnavus*; sequences attributed to *Escherichia* spp. all matched to *E. coli*.

### Reduction in CPT-11 toxicity by dietary fibres was not due to stimulation of beneficial bacterial groups

Beneficial effects of inulin and fructo-oligosaccharides are often attributed to stimulation of specific bacterial groups according to the concept of “prebiotics” [4]. To determine whether the influence of dietary fibre on CPT-11 toxicity can be attributed to their prebiotic effects, abundance of major bacterial groups was determined in samples obtained from experiments 2 and 3. Tables 1 and 2 depict abundance bacterial groups in animals fed different diets in Exp. 2 and 3, respectively. Additionally, the abundance of bacterial groups was correlated to all other parameters that were quantified in Exp. 2 and 3. heat maps depicting significant correlations in Exp. 2 and 3 are depicted in Figures 4 and 5, respectively. In Exp 2, only the abundance of the *Bacteroides* group (highest levels with FOS) and of the *Lactobacillus* group (highest levels with starch and resistant starch) differed among the seven diets (Table 1). However, none of the bacterial groups correlated to markers of animal health (Figure 4). Owing to the larger number of animals per group, Exp. 3 revealed more significant differences in intestinal microbiota of animals fed different dietary fibres (Table 2). β-Fructans increased abundance of lactobacilli and bifidobacteria but decreased abundance of the *Clostridium* cluster XI. However, none of the indicators of CPT-11 toxicity correlated to the abundance of specific bacterial groups. These results indicate that microbial ecology was altered by different fibre types; however, these changes did not mediate changes in CPT-11 toxicity.

### Reduction of toxicity by dietary fibres was associated with enhanced butyrate production

Whereas specific changes in composition of intestinal microbiota were unrelated to CPT-11 toxicity, activity of intestinal microbiota appeared to be a critical factor in CPT-11 toxicity. Cecal butyrate concentrations and feed intake were highly correlated in both experiments (Figure 4 and 5). Luminal concentrations of SCFA are a poor indicator of metabolic activity of intestinal microbiota because they represent a balance of microbial SCFA production and host absorption [23], therefore, abundance of genes coding for bacterial butyrate kinases and butyryl-CoA:acetate CoA-transferases as well as host expression MCT1 were additionally quantified. MCT1 expression had positive correlation with body weight and abundance of butyryl-CoA gene was positively correlated with butyrate concentration (Figure 5). Taken together, these correlations support the interpretation that the influence of dietary fibre on CPT-11 toxicity is partially mediated by an increased cecal production of butyrate.

## Discussion

Intestinal microbiota interact with the host digestive and immune systems. They can play multiple roles in various health conditions and either promote or prevent gut injury. The role of intestinal microbiota in CPT-11 chemotherapy has long been clearly, and yet restrictedly defined as toxicity activation through bacterial GUD, while other aspects have been overlooked. The present study provided a comprehensive view of four mechanisms in which gut microbiota may affect toxicity development during CPT-11/5-FU treatment, and explored the potential of using dietary fibres to reduce CPT-11 toxicity.

Bacterial translocation from the intestine can be promoted by microbial and/or host factors. This study identified translocating bacteria, and quantified abundance of translocating bacterial taxa in the gut lumen. Only three of 7 bacterial taxa increased moderately during chemotherapy and abundance of opportunistic pathogens remained low. CPT-11-chemotherapy impairs immune functions, i.e. depletion of cytotoxic T cells, and damages intestinal mucosa [22]. Although a localized increase in opportunistic pathogens at the mucosal surface cannot be ruled out, bacterial translocation during CPT-11 chemotherapy is likely caused mainly by host factors.

Inclusion of dietary fibre induced specific changes in gut microbiota. Overgrowth of *Clostridium* cluster XI is an indicator of intestinal dysbiosis [9]. Previously, *Clostridium* cluster XI was increased by both tumor and CPT-11 treatment [9] and reduced by inulin [24]. The reduced abundance of *Clostridium* cluster XI (∼5 logs) after inclusion of synergy in the diet was not accompanied by a noticeable difference in animal health. Bifidogenic effects of dietary fibres are considered a main mechanism by which fibres promote health [4], however, protective effects of commensal bifidobacteria were attributed primarily to the production of acetate [25]. The abundance of bifidobacteria after inclusion of synergy in the diet was higher than after inclusion of other dietary fibres. However, animal health was not correlated to the abundance of bifidobacteria.

GUD activity of intestinal microbiota in healthy animals allows the use of glucuronides as a carbon source for bacterial metabolism. Cell wall of plants and microbes contain glucuronides [26], [27], and xenobiotic compounds are conjugated to water-soluble glucuronides by liver UDP-glucuronosyltransferases and excreted with bile [28]. The release of xenobiotic compounds from glucuronides contributes to carcinogenesis in the lower gastrointestinal tract [29]. Effects of dietary fibres on GUD activity is specific for individual fibres [30], [31], [32]. Dietary effects on GUD activity are likely associated with group-specific changes in intestinal microbiota [32], the availability of energy source [33], as well as the availability of glucuronide substrate [34]. In this study, the primary determinant factor of GUD activity was the feed intake. Most rats showed substantial reduction in feed intake due to chemotherapy, reducing the availability of fermentable carbohydrates in the cecum and the overall activity of cecal microbiota. Quantification of SN-38G and SN-38 in jejunal and cecal digesta demonstrated that cecal GUD level was sufficient for complete deglucuronidation of SN-38 even in animals with low intestinal GUD activity. Strategies that target microbiota to reduce toxicity therefore require either drastic measures to suppress the entire flora (e.g. with antibiotics), or agents that specifically inhibit GUD activity [2]. In the present study, variation in SN-38 levels derived mainly from the upper gastrointestinal tract, most likely from hepatic metabolism, where dietary fibres have little impact. This also explains the large variations observed within treatments. The presence of GUD in intestinal bacteria is not as ubiquitous as other bacterial carbohydrate-active enzymes [20]. Major bacteria carriers of GUD were reported to be *Clostridium* cluster IV and XIVa [20]. In this study the most important producer was *Ruminococcus gnavus*. *R. gnavus* is a mucin-degrading species that increased disproportionately to total mucosa-associated bacteria in both Crohn's disease and ulcerative colitis [35]. *Enterbacteriaceae* accounted for less than 1% of total bacteria but also were significant contributors to intestinal GUD activity. Cecal *Enterobacteriaceae* were consistently increased by CPT-11-based regimens [9]. The role of *Enterobacteriaceae* in CPT-11 toxicity is thus three-fold: (i) their increased proportion in total bacteria reflects intestinal dysbiosis; (ii) they constituted the majority of bacteria that translocate across the intestinal barrier, and their lipopolysaccharide (LPS) can induce inflammatory responses and acute phase responses; (iii) they are important producers of GUD and may elevate SN-38 concentrations in the cecum.

Intestinal epithelial cells undergo considerable stress due to exposure to cytotoxic SN-38. This increases the requirement of energy for cellular repair and regeneration. Butyrate production is known as one of the key benefits of dietary fibres as it is an essential energy source of colonic epithelial cells [36]. However, the amount of substrate that is entering the large bowel and is available for microbial fermentation can be quantified in surgically modified swine models [37] but not in a rodent model as used in this study. Moreover, SCFA are rapidly absorbed by the host and the luminal concentrations represent only a fraction of the SCFA produced in microbial metabolism [37]. Therefore, three different indirect measurements were used. Quantification of luminal butyrate levels was complemented by quantification of the expression of monocarboxylate cotransporter 1 (MCT1), a H+-dependent symporter of butyrate. It is expressed in the gastrointestinal tract, with the highest expression in the cecum [38]. Its expression reflects the health of mucosal cells but it is also induced by luminal butyrate [19], [39], [40]. Butyrate production was also assessed by quantification of bacterial genes related to bacterial butyrate forming pathways. Butyrate formation is catalyzed by two alternative enzymes: butyrate kinase and butyryl-CoA transferase; the butyryl-CoA transferase pathway is predominant in adult human microbiota [12]. A reduction in butyryl-CoA transferase gene was reported for colorectal cancer [6] and Type I diabetes [41] and was considered a reflection of dysbiosis. However, primers used in the current study were designed for human microbiota, and may not amplify the corresponding genes of all butyrate producers in rodent microbiota [12]. In our study, abundance of the butyryl-CoA transferase gene outnumbered abundance of the butyrate kinase gene by one log in healthy rats, however, the combination of tumor and chemotherapy reversed this difference by reducing the butyryl-CoA transferase gene and increasing the butyrate kinase gene. Abundance of cecal butyryl-CoA transferase but not butyrate kinase was positively correlated with butyrate concentrations (Figure 5), consistent with the role of butyryl-CoA transferase as the major enzyme for butyrate production. Butyrate kinases have minor contribution to total butyrate production compared to butyryl-CoA transferases. The butyrate kinase pathway is employed by clostridia including *Cl. acetobutylicum*, *Cl. perfringens*, *Cl. tetani*, *Cl. botulinum* and *Cl. difficile* [12], of which many species are also gastrointestinal pathogens. Therefore, in contrast to butyryl-CoA transferase, whose abundance indicates the level of butyrate production, butyrate kinase contributes only a minor proportion to total butyrate production and is more likely associated with potentially harmful bacteria. Therefore, similar to *Clostridium cluster* XI and *Enterobacteriaceae* [9], its increase can be considered a sign of dysbiosis.

MCT1 expression was positively correlated with body weight, and cecal butyrate concentrations were correlated to feed intake in both experiments. Remarkably, other SCFAs were not associated with animal health and feed intake, which reflects the importance of colonic butyrate formation for the energy supply and function of intestinal mucosa [23], [42]. The specific association of butyrate and host health also indicates that this association is not merely a consequence of higher feed intake of healthy animals but indicates that stimulation of butyrate production by dietary fibre improves the condition of cecal and colonic mucosa and thus improves barrier properties and immune function. Despite the strong correlation between butyrate production and host well-being, a clear diet effect on host health was not observed for any type of fibre. The variations in the hosts' metabolism of CPT-11 were large when compared to the modest effect of dietary fibre. A second possible reason could be the low dosage of fibre used. The quantitative assessment of starch digestion in swine revealed that inclusion of 40% of RS in diet is required to substantially increased colonic butyrate formation [37]. Our experimental diet contained 8% w/w fibre, which was marginal to produce any discernible difference.

In conclusion, this study demonstrates for the first time that dietary modulation by optimizing butyrate production reduces irinotecan-induced toxicity. This finding may be explored to reduce morbidity, and to improve tolerance of tolerability irinotecan chemotherapy.

## Supporting Information

Table S1 **Dietary composition for Experiments 2 and 3. Data from Farhangfar** [**22**] **.**
(DOCX)

Table S2 **Taxonomic identification of translocated bacteria isolated from mesenteric lymphnodes in two CPT-11-based regimens.**
(DOCX)
