# Supplementary material for: The Role of Intestinal Microbiota in Development of Irinotecan Toxicity and in Toxicity Reduction through Dietary Fibres in Rats
Source: PLoS One. 2014 Jan 14;9(1):e83644. doi: 10.1371/journal.pone.0083644 (PMC3891650; doi:10.1371/journal.pone.0083644)
Supplement: Table S1 — Dietary composition for Experiments 2 and 3. Data from Farhangfar [22] . (DOCX) [file pone.0083644.s001.docx]

**Lin et al. Online Supplementary Information**

**Table S1.** Dietary composition for Experiments 2 and 3. Data from Farhangfar [22].

| **Ingredients [g]** | | | | | | | | |
| --- | --- | --- | --- | --- | --- | --- | --- | --- |
| **Variable portion (8%)** | | | | | | | | |
|  |  | Cell. | Starch | IMO | FOS | In. | Syn | RS |
| **Non digestible carbohydrates** | Cellulose | 8 | 0 | 0 | 0 | 0 | 0 | 0 |
|  | Cornstarch | 0 | 8 | 0 | 0 | 0 | 0 | 0 |
|  | Isomalto-oligosacchrides | 0 | 0 | 8 | 0 | 0 | 0 | 0 |
|  | Fructo-oligosaccharides | 0 | 0 | 0 | 8 | 0 | 0 | 0 |
|  | Inulin | 0 | 0 | 0 | 0 | 8 | 0 | 0 |
|  | Synergy | 0 | 0 | 0 | 0 | 0 | 8 | 0 |
|  | Resistant starch | 0 | 0 | 0 | 0 | 0 | 0 | 8 |
| **Constant portion (92%)** | | | | | | | | |
| **Modified AIN-76 basal mix** | Casein (25.2g), methionine (0.25g), glucose (13.95g), vitamins AIN 76 (1g), minerals AIN 76 (5g), inositol (0.6g), cornstarch (23.7g), cellulose 2 g | 72 | 72 | 72 | 72 | 72 | 72 | 72 |
| **Lipids** | canola stearine (11.4g), linseed oil (0.8g), sunflower oil (7.8g) | 20 | 20 | 20 | 20 | 20 | 20 | 20 |
| Total |  | 100 | 100 | 100 | 100 | 100 | 100 | 100 |
