# Supplementary material for: The Role of Intestinal Microbiota in Development of Irinotecan Toxicity and in Toxicity Reduction through Dietary Fibres in Rats
Source: PLoS One. 2014 Jan 14;9(1):e83644. doi: 10.1371/journal.pone.0083644 (PMC3891650; doi:10.1371/journal.pone.0083644)
Supplement: Table S2 — Taxonomic identification of translocated bacteria isolated from mesenteric lymphnodes in two CPT-11-based regimens. (DOCX) [file pone.0083644.s002.docx]

**Lin et al. Online Supplementary Information**

**Table S2**. Taxonomic identification of translocated bacteria isolated from mesenteric lymphnodes in two CPT-11-based regimens.

|  | **Sequence Accession number** | **# of Base Pairs Sequenced** | **Homology to type strain** |
| --- | --- | --- | --- |
| *Morganella morganii* FUA1235 | HQ169114 | 1455 | 0.971 |
| *Escherichia coli* FUA1236 | HQ169115 | 612 | 1.000 |
| *Proteus mirabilis* FUA1237 | HQ169116 | 1454 | 0.982 |
| *Proteus mirabilis* FUA1239 | HQ169117 | 1455 | 0.983 |
| *Proteus mirabilis* FUA1240 | HQ169118 | 1454 | 0.979 |
| *Staphylococcus epidermidis* FUA2058 | HQ169119 | 1464 | 0.999 |
| *Enterococcus avium* FUA3332 | HQ169120 | 1471 | 1.000 |
| *Staphylococcus cohnii* FUA2059 | HQ169121 | 1464 | 0.981 |
| *Escherichia coli* FUA1241 | HQ169122 | 1455 | 1.000 |
| *Enterococcus faecalis* FUA3333 | HQ169123 | 653 | 1.000 |
| *Escherichia coli* FUA1242 | HQ169124 | 1454 | 0.998 |
| *Morganella morganii* FUA1243 | HQ169125 | 1454 | 0.989 |
| *Enterococcus faecalis* FUA 3334 | HQ184922 | 1475 | 0.976 |
| *Morganella morganii* FUA1245 | HQ169126 | 1455 | 0.983 |
| *Citrobacter freundii* | HQ694731 | 1408 | 0.976 |
| *Klebsiella oxytoca* | HQ694732 | 1403 | 0.983 |
| *Proteus mirabilis* | HQ694733 | 1267 | 0.992 |
| *Staphylococcus warneri* | HQ694734 | 1301 | 1.000 |
